# Adipose-Derived Mesenchymal Stem Cells Isolated from Patients with Abdominal Aortic Aneurysm Exhibit Senescence Phenomena

**DOI:** 10.1155/2019/1305049

**Published:** 2019-11-25

**Authors:** Xiaoran Huang, Hao Zhang, Xiaoting Liang, Yimei Hong, Mengmeng Mao, Qian Han, Haiwei He, Wuyuan Tao, Guojun Jiang, Yuelin Zhang, Xin Li

**Affiliations:** ^1^Medical College, Shantou University, Shantou, Guangdong, China; ^2^Department of Emergency Medicine, Department of Emergency and Critical Care Medicine, Guangdong Provincial People's Hospital, Guangdong Academy of Medical Sciences, Guangzhou, Guangdong, China; ^3^School of Pharmacy, Bengbu Medical College, Bengbu, Anhui, China; ^4^Clinical Translational Medical Research Centre, Shanghai East Hospital, Tongji University School of Medicine, Shanghai, China; ^5^Department of Medicine, The First Affiliated Hospital of Guangzhou Medical University, Guangzhou Institute of Respiratory Health, State Key Laboratory of Respiratory Disease, Guangzhou, Guangdong, China

## Abstract

Mesenchymal stem cells (MSCs) have shown beneficial effects in the treatment of abdominal aortic aneurysm (AAA). Nonetheless, the biological properties of adipose-derived MSCs (ASCs) from patients with AAA (AAA-ASCs) remain unclear. This study is aimed at investigating the properties of cell phenotype and function of AAA-ASCs compared with ASCs from age-matched healthy donors (H-ASCs). H-ASCs and AAA-ASCs were studied for cell phenotype, differentiation capacity, senescence, and mitochondrial and autophagic functions. Cellular senescence was examined by senescence-associated *β*-galactosidase (SA-*β*-gal) staining. Mitochondrial morphology was determined by MitoTracker staining. Despite the similar surface markers of AAA-ASCs and H-ASCs, AAA-ASCs exhibited altered multidifferentiation potential. Compared with H-ASCs, AAA-ASCs displayed enhanced senescence manifested by increased SA-*β*-gal activity and decreased proliferation and migration ability. Furthermore, AAA-ASCs showed increased mitochondrial fusion, reactive oxygen species (ROS) production, and decreased mitochondrial membrane potential. In addition, AAA-ASCs exhibited decreased autophagy level, upregulation of IL-6 and TNF-*α* secretion, and downregulation of IL-10 secretion compared with H-ASCs. Nonetheless, treatment of AAA-ASCs with rapamycin (an autophagy activator) dramatically reduced secretion of IL-6 and TNF-*α* and enhanced secretion of IL-10. In conclusion, our study showed that AAA-ASCs exhibit senescence phenomena and decreased cell function. Understanding the specific alterations in AAA-ASCs will help explore novel strategies to restore cell function for AAA treatment.

## 1. Introduction

Abdominal aortic aneurysm (AAA), which is characterized by loss of vascular smooth muscle cells, extracellular matrix (ECM) degradation, and progressive abdominal aortic dilation, is the leading cause of morbidity and mortality in the elderly [1]. The incidence of AAA in the aging population is estimated to be 6 to 9% [2]. Although the underlying mechanisms are not fully understood, chronic inflammation of the arterial wall is known to play a critical role in regulating its pathogenesis [3–5]. Despite the advances in pharmacological strategies including macrolides, tetracyclines, statins, and surgical interventions, there remains no effective treatment to prevent AAA progression and rupture [6]. Identifying novel strategies to prevent AAA is urgently needed.

Mesenchymal stem cells (MSCs) are multipotent cells that can differentiate into a variety of cell types including adipocytes, osteoblasts, and chondrocytes. There is accumulating evidence of the benefit of MSCs as cellular therapy for various inflammation-related diseases owing to their unique immunomodulatory properties [7–9]. Indeed, over the past decades, MSC-based therapy has emerged as a promising approach for AAA treatment. Administration of human MSCs protects against AAA formation by inhibiting CD4^+^ T-cell-produced proinflammatory cytokines [10]. Transplantation of MSCs into apoE(-/-) mice has been shown to attenuate Ang II-induced AAA formation by downregulating levels of MMPs and inflammatory cytokines and restoring elastin in the aortic wall [11]. Currently, MSCs can be obtained from many adult tissues, the most common being bone marrow (BM) and adipose tissue [12]. Nonetheless, their isolation from BM is limited by the number of cells obtained and the need for an invasive procedure [13]. In contrast, adipose tissue is widely distributed in the human body and contains a larger number of MSCs. Nevertheless, the function of adipose-derived MSCs (ASCs) isolated from patients greatly declines. ASCs from patients with coronary artery disease exhibit a decreased immunomodulatory capacity due to excessive reactive oxygen species (ROS) production [14] although the biological properties of those from patients with AAA (AAA-ASCs) have not been determined. In the current study, we assessed the phenotype and functionality of AAA-ASCs and ASCs from age-matched healthy donors (H-ASCs).

## 2. Materials and Methods

### 2.1. Study Subjects

This study was approved by the research ethics board of Guangdong Provincial People's Hospital, China. All participants provided written informed consent, and the demographic information is summarized in Table 1. Subcutaneous adipose tissue was harvested from 13 AAA patients undergoing surgery and 11 age-matched healthy donors.

### 2.2. Isolation and Characterization of ASCs

ASCs were isolated from adipose tissue from healthy donors and AAA patients as previously reported [15]. Briefly, adipose tissue (1-5 g) was cut into small pieces and digested with enzyme and subsequently plated on 10 cm culture dishes. After 48 hours, nonadherent cells were washed off and the remaining cells cultured with DMEM/low glucose (Gibco) medium supplemented with 10% FBS (Life Technologies, 16000), 0.1 mM 2-mercaptoethanol (Life Technologies, 21985023), NEAA (Life Technologies, 11140050), and 0.1% Penicillin/Streptomycin (Life Technologies, 15140122). The ASCs at passages 3~4 were used in the current study. Both H-ASCs and AAA-ASCs were passaged at 3-day intervals and the same cell number (100,000 cells per 6 cm dish) plated. Population doubling was evaluated at each passage.

Surface markers of H-ASCs and AAA-ASCs were determined using flow cytometry. Antibodies including anti-CD31 (BioLegend, 303111), anti-CD45 (BioLegend, 304011), anti-CD73 (BioLegend, 344003), anti-CD90 (BioLegend, 328107), and anti-CD105 (BioLegend, 323205) were used. The differentiation capacity of H-ASCs and AAA-ASCs into adipocytes and osteocytes was examined as previously described [16].

### 2.3. Senescence-Associated *β*-Galactosidase (SA-*β*-Gal) Staining

The cellular senescence of ASCs was determined using a SA-*β*-gal staining kit (Beyotime, C0602) according to the manufacturer's protocol. Briefly, the same number of H-ASCs and AAA-ASCs after passage was plated on a 6-well plate. After 24 hours, ASCs were washed with PBS, fixed with fixative solution for 15 minutes, and subsequently incubated with SA-*β*-gal staining solution overnight at 37°C in an incubator without CO_2_. Finally, the senescent ASCs with blue color were captured from five different view fields of each sample and their senescence analyzed.

### 2.4. Immunofluorescence Staining

Immunofluorescence staining was performed as previously described [17]. H-ASCs and AAA-ASCs were cultured in a 24-well plate with glass cover slides. After fixation with formaldehyde for 30 minutes, H-ASCs and AAA-ASCs were permeated with 0.1% Triton X-100 in PBS for 30 minutes and then incubated with Ki67 antibody (1 : 100, Abcam, ab15580) and *γ*H2AX antibody (1 : 100, Abcam, ab81299) overnight at 4°C. Next, ASCs were incubated with the fluorescent-labeled secondary antibodies for 1 hour at room temperature in the dark and then mounted with 4′,6-diamidino-2-phenylindole (DAPI; Vector Laboratories, Inc.) to stain the nucleus. Five view fields from each slide were randomly photographed using a fluorescent microscope.

### 2.5. Scratch-Wound Assay

H-ASCs and AAA-ASCs were plated on a 12-well plate and cultured until they reached 90% confluence. The same-width scratches were made using a 1 mL pipette tip on the bottom of the plate. Next, H-ASCs and AAA-ASCs were gently washed with PBS and then cultured with medium without serum in an incubator with 5% CO_2_ at 37°C. After 24 hours of incubation, the migration of ASCs into the “wound” area was captured using a phase contrast microscope and analyzed.

### 2.6. MitoTracker Staining

The mitochondrial morphology of ASCs was evaluated by MitoTracker Green FM (Invitrogen, M7514). Briefly, H-ASCs and AAA-ASCs were incubated with DMEM supplemented with 20 nM/L MitoTracker Green FM in the dark for 30 minutes. Subsequently, cells were mounted with DAPI and photographed under a confocal microscope.

### 2.7. ROS Measurement

Mitochondrial ROS in ASCs was measured by MitoSOX staining (Invitrogen, M36008). Briefly, H-ASCs and AAA-ASCs were cultured in a 24-well plate with glass cover slides until 70~80% confluence. After washing with PBS three times, H-ASCs and AAA-ASCs were cultured with 5 *μ*mol/L MitoSOX at 37°C for 15 minutes in the dark. Images of five different view fields of H-ASCs and AAA-ASCs from each slide were randomly captured. The fluorescence intensity was calculated using ImageJ software (National Institutes of Health, Bethesda, MD, USA) in three independent experiments.

### 2.8. JC-1 Staining

The mitochondrial membrane potential (MMP) was determined by JC-1 dye (Thermo Fisher Scientific, T3168) according to the manufacturer's protocol. Briefly, H-ASCs and AAA-ASCs were cultured in 24-well plates with glass coverslips and then stained with JC-1 dye for 10 minutes. Finally, five different view fields of H-ASCs and AAA-ASCs were randomly photographed and fluorescence intensity calculated using ImageJ software in three independent experiments.

### 2.9. Western Blotting

The proteins of H-ASCs and AAA-ASCs were extracted and their concentration determined using a bicinchoninic acid assay kit (Thermo Fisher Scientific, 231227). A total of 25 *μ*g of protein of each sample was loaded, separated by SDS/PAGE, and then transferred to PVDF membranes. After blocking with 5% fat-free milk in TBST, the membranes were incubated at 4°C overnight with the following antibodies: anti-p-Drp1 ser616 (1 : 1000, Invitrogen, PA5-64821), anti-Drp1 (1 : 1000, Invitrogen, PA5-20176), anti-Mfn2 (1 : 1000, Abcam, ab124773), anti-p53 (1 : 1000, Abcam, ab26), anti-p21 (1 : 1000, Abcam, ab109199), anti-Beclin (1 : 1000, CST, 3738), anti-LC3I/II (1 : 1000, CST, 4108), anti-p62 (1 : 1000, CST, 5114), and GAPDH (1 : 1000, CST, 2118). Subsequently, after washing with TBST three times, the membranes were incubated with secondary antibodies (1 : 3000, CST) at room temperature for 1 hour and then exposed in a dark room.

### 2.10. Transmission Electron Microscope (TEM)

Examination of ASCs using a TEM was performed as previously described [18]. Briefly, after washing with PBS, the cells were fixed with 2.5% glutaraldehyde in phosphate buffer for 4 h and then postfixed with 1% OsO_4_ for 2 h. Next, cells were dehydrated with a graded concentration of ethanol (30, 50, 70, 80, 90, 95, and 100%). Subsequently, cells were infiltrated with 1 : 1 acetone : Spurr resin (SPI-Chem, 02690-AB) for 1 h at room temperature, 1 : 3 acetone : Spurr resin for 3 h, and then absolute Spurr resin overnight. Electron images were captured under a TEM (Hitachi, H-7650).

### 2.11. Enzyme-Linked Immunosorbent Assay (ELISA)

Conditioned medium from H-ASCs, AAA-ASCs, or rapamycin-treated AAA-ASCs was prepared as previously reported [19]. The concentration of inflammation-related cytokines, including IL-6, IL-10, and TNF-*α*, in the conditioned medium was detected by ELISA. Each experiment was repeated three times.

### 2.12. ATP Content Measurement

ATP in H-ASCs and AAA-ASCs was harvested with boiling distilled water as previously reported [20]. The ATP content was examined using an ATP Determination Kit (Molecular Probes, A22066).

### 2.13. Telomere Length Measurement

Genomic DNA was extracted directly from H-ASCs and AAA-ASCs using standard procedures. The relative telomere length was determined as previously described [21]. Briefly, the telomere length was represented by the relative ratio of the telomere repeat copy number (*T*) to the single-copy gene 36B4 copy number (*S*). The *T*/*S* ratio was determined by quantitative polymerase chain reaction (qPCR) using a 7900HT thermal cycler (Applied Biosystems). It can then be calculated by the formula *T*/*S* = 2^(−dCt)^, where dCt is the difference in threshold cycle obtained by subtracting the average 36B4 Ct value from the average telomere Ct value. Primer sequences are as follows: Tel 1b—270 nM, 5′-GGTTTTTGAGGGTGAGGGTGAGGGTGAGGGTGAGGGT-3′; Tel 2b—900 nM, 5′-TCCCGACTATCCCTATCCCTATCCCTATCCCTATCCCTA-3′, 36B4u—300 nM, 5′-CAGCAAGTGGGAAGGTGTAATCC-3′; and 36B4d—500 nM, 5′-CCCATTCTATCATCAACGGGTACAA-3′. All samples were measured in triplicate.

### 2.14. Statistical Analysis

All values are expressed as mean ± SEM. Statistical analyses were performed using Prism 5.04 Software (GraphPad Software for Windows, San Diego, CA, USA). Comparison between two groups was analyzed by unpaired Student's *t*-test. Comparison between multiple groups was analyzed by using one-way ANOVA followed by the Bonferroni test. A *p* < 0.05 was considered statistically significant.

## 3. Results

### 3.1. Characterization of H-ASCs and AAA-ASCs

**Figure 1:**
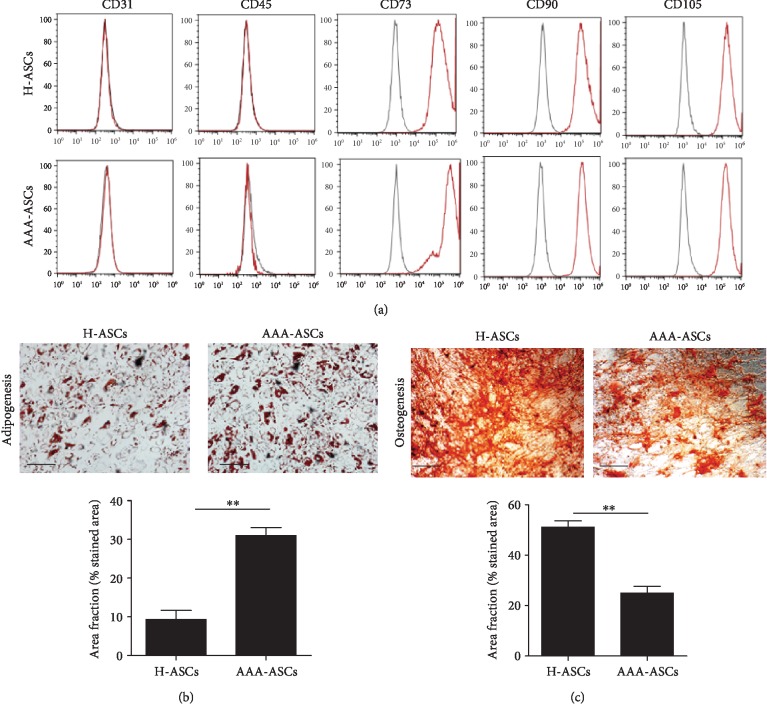
Characterization of H-ASCs and AAA-ASCs. (a) The surface markers of H-ASCs and AAA-ASCs were examined by flow cytometry. Both YMSCs and AMSCs were positive for the MSC-specific markers CD73, CD90, and CD105 but negative for CD31 and CD45. (b) Adipogenic differentiation evaluated by Oil Red O staining and quantification of adipogenic efficiency in H-ASCs and AAA-ASCs. Scale bar = 200 *μ*m. (c) Osteogenic differentiation evaluated by Alizarin Red staining and quantification of osteogenic efficiency in H-ASCs and AAA-ASCs. Scale bar = 200 *μ*m. Data are expressed as mean ± SEM. *n* = 3. ^∗∗^*p* < 0.01.

We evaluated the surface antigens of H-ASCs and AAA-ASCs using flow cytometry. The results showed that H-ASCs and AAA-ASCs expressed similar surface markers including negative for CD31 and CD45 and positive for CD73, CD90, and CD105 (Figure 1(a)). Subsequently, we examined the differentiation capacity of H-ASCs and AAA-ASCs into adipocytes and osteocytes. As shown in Figure 1(b), both H-ASCs and AAA-ASCs differentiated into adipocytes and osteocytes as manifested by Oil Red O staining and Alizarin Red staining (Figures 1(b) and 1(c)). Notably, quantification of Oil Red O staining showed a great increase in the percentage positive area, whereas Alizarin Red staining showed a decreased percentage of positive area in AAA-ASCs after differentiation compared with H-ASCs, suggesting that the differentiation capacity of AAA-ASCs was altered (Figures 1(b) and 1(c)).

### 3.2. AAA-ASCs Are More Senescent than H-ASCs

**Figure 2:**
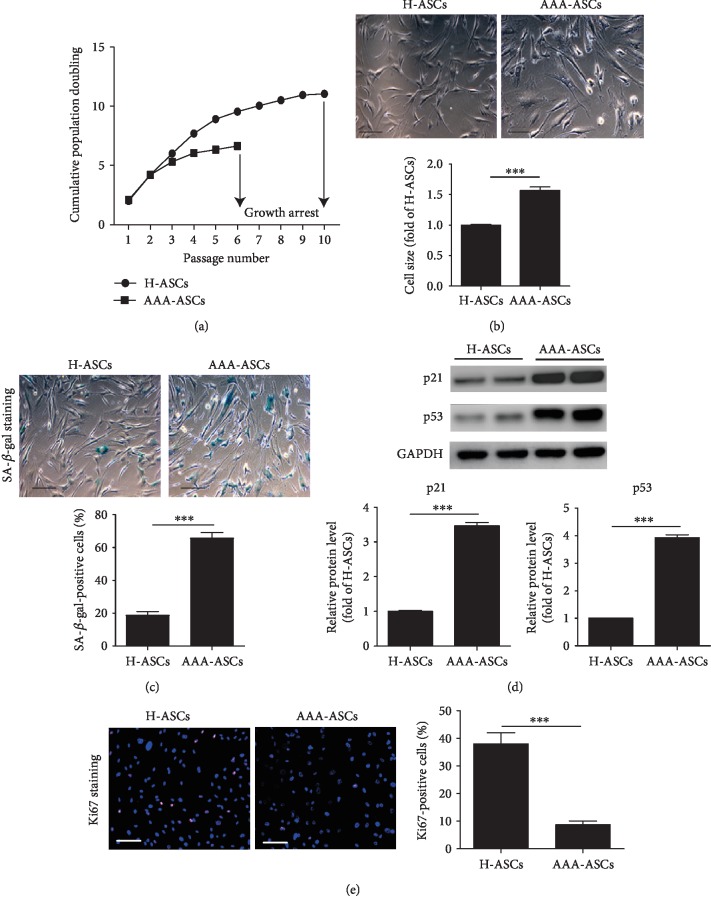
AAA-ASCs displayed increased cellular senescence. (a) Cell growth curves show the lower proliferative ability of AAA-ASCs compared to that of H-ASCs. (b) Representative images of cell morphology and quantitative analysis of cell size in H-ASCs and AAA-ASCs. Scale bar = 200 *μ*m. (c) Representative images of SA-*β*-gal staining and quantitative analysis of SA-*β*-gal-positive cells in H-ASCs and AAA-ASCs. Scale bar = 200 *μ*m. (d) Western blotting and quantitative analysis of the expression levels of p53 and p21 in H-ASCs and AAA-ASCs. (e) Immunostaining of the proliferation marker Ki67 and quantitative analysis of Ki67-positive cells in H-ASCs and AAA-ASCs. Scale bar = 200 *μ*m. Data are expressed as mean ± SEM. *n* = 3. ^∗∗∗^*p* < 0.001.

We examined the cell growth rate of H-ASCs and AAA-ASCs via serial passaging. As shown in Figure 2(a), AAA-ASCs demonstrated a lower growth rate and arrested growth at passage 6, whereas H-ASCs continued growing until passage 10, suggesting that the proliferative capacity of AAA-ASCs was decreased (Figure 2(a)). AAA-ASCs exhibited increased cell size compared with H-ASCs (Figure 2(b)). Next, we performed SA-*β*-gal staining to determine the cellular senescence of H-ASCs and AAA-ASCs. Compared with H-ASCs, the percentage of SA-*β*-gal-positive cells was dramatically increased in AAA-ASCs (Figure 2(c)). Furthermore, the protein level of cellular senescence markers such as p53 and p21 was also significantly increased in AAA-ASCs compared with those in H-ASCs (Figure 2(d)). In addition, Ki67 staining showed that the proliferative rate of AAA-ASCs was much lower than that of H-ASCs (Figure 2(e)). These data suggest that AAA-ASCs are more senescent than H-ASCs.

### 3.3. AAA-ASCs Exhibit Increased DNA Damage and Decreased Migration Capacity

**Figure 3:**
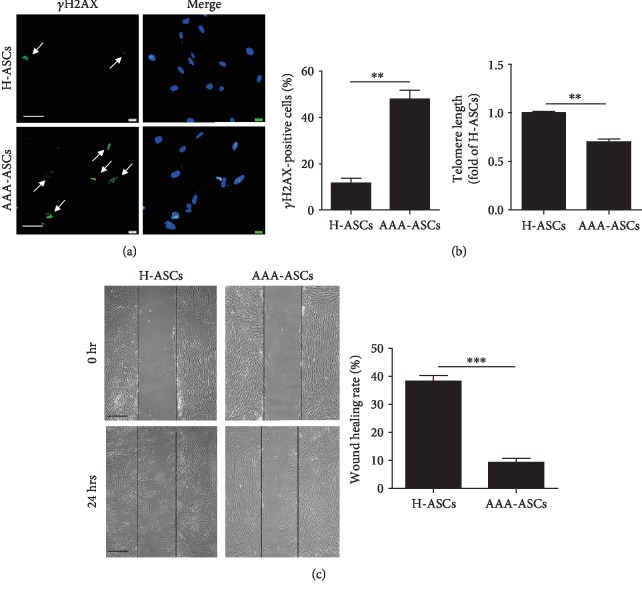
AAA-ASCs demonstrated increased DNA damage and decreased migration capacity. (a) Representative images of *γ*H2AX staining and quantitative analysis of *γ*H2AX-positive cells in H-ASCs and AAA-ASCs. Scale bar = 50 *μ*m. (b) Quantitative analysis of the telomere length in H-ASCs and AAA-ASCs. (c) Representative images of scratches of wound healing demonstrating the migration ability and quantification of the wound recovery rate of H-ASCs and AAA-ASCs. Scale bar = 200 *μ*m. Data are expressed as mean ± SEM. *n* = 3. ^∗∗^*p* < 0.01; ^∗∗∗^*p* < 0.001.

It has been reported that the accumulation of DNA damage is a leading cause of cellular senescence [22]. We examined DNA damage in AAA-ASCs and H-ASCs using *γ*H2AX staining. The percentage of *γ*H2AX-positive cells was significantly enhanced in AAA-ASCs compared with H-ASCs (Figure 3(a)). We also measured the telomere length in AAA-ASCs and H-ASCs. AAA-ASCs exhibited a shorter telomere length than H-ASCs (Figure 3(b)). Next, we evaluated the migratory capacity of AAA-ASCs and H-ASCs using scratch assays. Both AAA-ASCs and H-ASCs invaded the scratch area within 24 hours (Figure 3(c)). Notably, AAA-ASCs presented reduced migration into the scratch area compared with H-ASCs (Figure 3(c)).

### 3.4. Mitochondrial Function Is Impaired in AAA-ASCs

**Figure 4:**
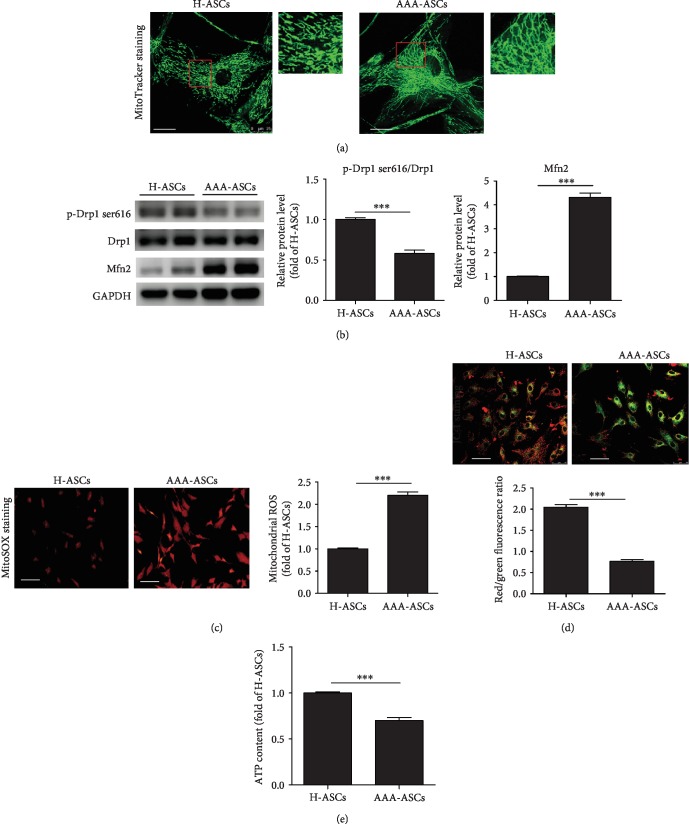
Mitochondrial function was decreased in AAA-ASCs. (a) Representative images of mitochondrial morphology determined by MitoTracker staining in H-ASCs and AAA-ASCs. Scale bar = 25 *μ*m. (b) Western blotting and quantitative analysis of the expression level of p-Drp1 ser616/Drp1 and Mfn2 in H-ASCs and AAA-ASCs. (c) Representative images of ROS determined by MitoSOX staining and quantitative analysis of ROS generation in H-ASCs and AAA-ASCs. Scale bar = 100 *μ*m. (d) Representative images of MMP determined by JC-1 staining and quantitative analysis of MMP in H-ASCs and AAA-ASCs. (e) The intracellular ATP level in H-ASCs and AAA-ASCs. Data are expressed as mean ± SEM. *n* = 3. ^∗∗∗^*p* < 0.001.

Accumulating evidence has shown that mitochondrial dysfunction plays a critical role in regulating cellular senescence [23–25]. Therefore, we examined the mitochondrial function in AAA-ASCs. It has been reported that mitochondrial morphology is closely associated with mitochondrial function [26]. First, we examined the morphology of mitochondria in AAA-ASCs and H-ASCs. MitoTracker staining results showed an increased mitochondrial length in AAA-ASCs (Figure 4(a)). Western blotting showed that compared with H-ASCs, the level of mitochondrial fission protein p-Drp-1 ser616 was significantly reduced in AAA-ASCs, whereas the level of mitochondrial fusion protein Mitofusin 2 (Mfn2) was greatly increased (Figure 4(b)), suggesting increased mitochondrial fusion in AAA-ASCs. Disruption of mitochondrial dynamics contributes to mitochondrial ROS generation. We evaluated ROS generation in AAA-ASCs and H-ASCs using MitoSOX staining and revealed it to be significantly increased in AAA-ASCs compared with H-ASCs (Figure 4(c)). Subsequently, we examined mitochondrial membrane potential (MMP, ΔΨ*m*) using JC-1 staining in AAA-ASCs and H-ASCs. The JC-1 signal (red color, aggregates, high potential; green color, monomers, low potential) displays the ΔΨ*m* in the mitochondria. The results showed a significant reduction in the red/green fluorescence ratio in AAA-ASCs compared with H-ASCs (Figure 4(d)), indicating the ΔΨ*m* collapse in AAA-ASCs. Furthermore, the intracellular ATP level in AAA-ASCs was significantly reduced compared with H-ASCs (Figure 4(e)). Collectively, these data show that mitochondrial function is impaired in AAA-ASCs.

### 3.5. Autophagy Level Is Decreased in AAA-ASCs

**Figure 5:**
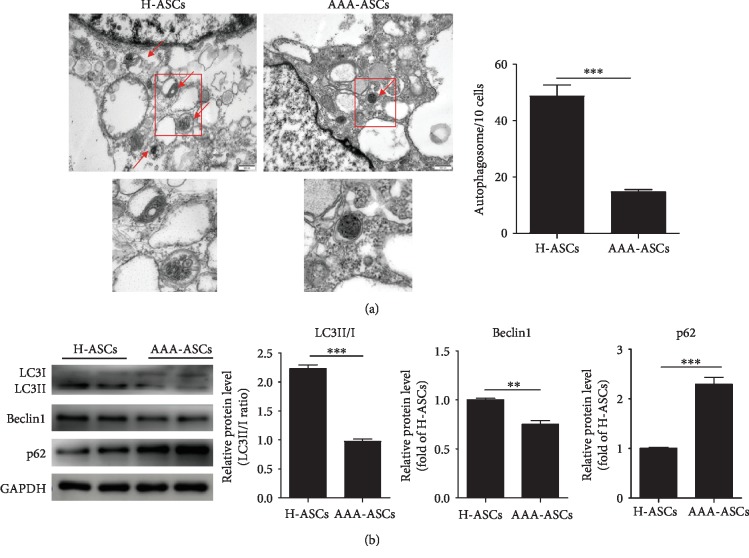
Autophagy level was decreased in AAA-ASCs. (a) Representative images of autophagosomes examined by a TEM and quantitative analysis of autophagosomes in H-ASCs and AAA-ASCs. Scale bar = 500 nm. (b) Western blotting and quantitative analysis of the expression level of LC3II/I, Beclin, and p62 in H-ASCs and AAA-ASCs. Data are expressed as mean ± SEM. *n* = 3. ^∗∗^*p* < 0.01; ^∗∗∗^*p* < 0.001.

Growing evidence has shown that autophagy mediates the cellular senescence of MSCs [27, 28]. We first detected the autophagosomes in each cell using a TEM. As shown in Figure 5(a), the number of autophagosomes in AAA-MSCs was much lower than that in H-ASCs (Figure 5(a)). To verify this, we detected the protein expression of some key autophagy-associated proteins LC3II/I, Beclin, and p62. Compared with H-ASCs, the protein expression of LC3II/I and Beclin was significantly downregulated in AAA-MSCs, whereas the level of p62 protein was upregulated (Figure 5(b)). These results suggest that the autophagy level is decreased in AAA-ASCs.

### 3.6. AAA-ASCs Display an Altered Secretome

**Figure 6:**
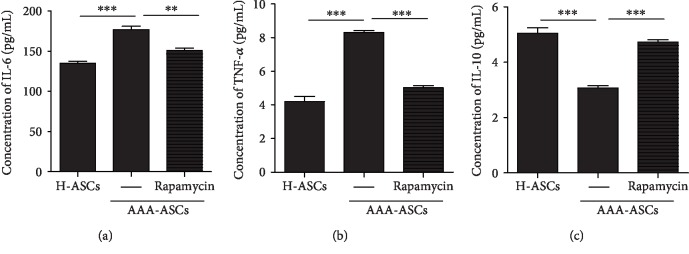
Changes in the secretion of inflammatory factors in AAA-ASCs. (a) Concentration of IL-6 in medium conditioned by H-ASCs, AAA-ASCs, or AAA-ASCs treated with rapamycin. (b) Concentration of TNF-*α* in medium conditioned by H-ASCs, AAA-ASCs, or AAA-ASCs treated with rapamycin. (c) Concentration of IL-10 in medium conditioned by H-ASCs, AAA-ASCs, or AAA-ASCs treated with rapamycin. Data are expressed as mean ± SEM. *n* = 3.^∗∗^*p* < 0.01; ^∗∗∗^*p* < 0.001.

The MSC secretome plays an important role in the regulation of MSC immunopotency. Because autophagy promotes the paracrine effects of MSCs [29], we evaluated key immunomodulatory cytokines in the H-ASC and AAA-MSC secretome. AAA-MSCs secreted higher levels of IL-6 and TNF-*α* and a lower level of IL-10 than H-ASCs (Figures 6(a)–6(c)). Moreover, treatment of AAA-ASCs with the autophagy activator, rapamycin, significantly reduced the levels of IL-6 and TNF-*α* and increased the level of IL-10 in the secretome, suggesting that autophagy may play a role in regulation of the immunomodulatory properties of AAA-ASCs (Figures 6(a)–6(c)).

## 4. Discussion

There were several major findings in the current study. First, ASCs derived from AAA patients displayed phenomena of senescence, manifested by a decreased proliferative capacity, increased SA-*β*-gal activity, and DNA damage. Second, AAA-ASCs exhibited increased mitochondrial fusion and decreased mitochondrial fission, leading to excessive ROS production and reduced MMP. Third, the autophagy level of AAA-ASCs was decreased. Fourth, AAA-ASCs secreted higher levels of the inflammatory cytokines IL-6 and TNF-*α* and a lower level of IL-10, an effect partially reversed by rapamycin treatment.

A growing body of evidence from preclinical studies and clinical trials has shown the promising results of autologous ASC transplantation in various disorders including cardiovascular diseases and AAA [30–33]. Nevertheless, the function of ASCs is adversely influenced by a patient's comorbid conditions, thus affecting their therapeutic efficiency. In the current study, we successfully isolated ASCs from healthy age-matched controls and AAA patients. Although we observed no difference in the surface antigen profile between AAA-ASCs and H-ASCs, the differentiation capacity of AAA-ASCs into adipocytes was dramatically increased while that into osteocytes was decreased, indicating that the differentiation tendency of AAA-ASCs was modified. These findings are consistent with a previous study showing that adipose-derived mesenchymal stromal cells from diabetic patients exhibit an altered differentiation tendency [34]. It has been reported that the function of MSCs declines with aging [35]. Aging MSCs tend to lose their differentiation capacity. These results promote us to speculate that the altered differentiation capacity of AAA-ASCs may be caused by cellular senescence. As expected, we found that SA-*β*-gal activity and protein levels of p21 and p53, hallmarks of aging, were significantly increased in AAA-ASCs compared with those in H-ASCs, demonstrating the cellular senescence of AAA-ASCs. Furthermore, the proliferative and migratory capacities were also decreased in AAA-ASCs. These data provide further evidence that AAA-ASCs present impaired function. Nevertheless, the potential mechanisms underlying AAA-ASC senescence remain to be elucidated.

To the best of our knowledge, mitochondria are dynamic organelles that undergo fission and fusion. Mitochondrial fusion is mainly mediated by Mfn1/2 and optic atrophy protein 1 (OPA1). Mitochondrial fission is regulated by Drp1 and fission-1 (Fis1). Recent studies have highlighted that abnormal mitochondrial dynamics are related to cellular senescence [36, 37]. Our previous study also showed that MSCs display increased mitochondrial fusion as evidenced by the upregulation of Mfn2 and the downregulation of p-Dp1 ser616 accompanied by increased cellular senescence during their expansion *in vitro* [23]. Similarly, we found that AAA-ASCs exhibited increased mitochondrial length and increased protein level of Mfn2 and decreased protein level of p-Drp1 ser616, suggesting that mitochondrial fusion occurs in AAA-ASCs. In contrast, the senescent MSCs derived from idiopathic pulmonary fibrosis patients had fragmented and dysfunctional mitochondria [38]. The different mitochondrial morphology may be due to the variety of patients with different comorbid conditions. Mitochondrial morphology is inherently associated with function. Mitochondrial fusion in MSCs leads to increased ROS generation and a reduced MMP, inducing mitochondrial dysfunction [14]. We further showed that AAA-ASCs had elevated mitochondrial ROS levels and decreased MMP. Since mitochondrial dysfunction plays a critical role in AAA-ASC senescence, it is worth investigating whether the restoration of mitochondrial dysfunction can rejuvenate AAA-ASCs.

In addition to mitochondrial dysfunction, we observed that the level of autophagy was reduced in AAA-ASCs. Indeed, the basal level of autophagy contributes to maintain cell function including survival, differentiation, and homoeostasis under different conditions [39]. Increasing evidence has shown that altered autophagic activity is a major cause of cellular senescence [40, 41]. A previous study has demonstrated that the downregulation of autophagy enhances ROS generation and p53 levels, leading to MSC senescence, whereas activating autophagy by rapamycin improves the function of aged MSCs [35]. Hypoxia treatment, via the regulation of the HIF1*α*/AIMP3 pathway, delays MSC senescence by inducing autophagy [27]. In the current study, the protein level of Beclin1 and LC3II/I was significantly reduced, whereas that of p62 was increased in AAA-ASCs compared with H-ASCs, suggesting defective autophagy in AAA-ASCs. Therefore, a defective autophagy might contribute to AAA-ASC senescence. Nevertheless, the potential link between AAA-ASC senescence and autophagy remains unclear.

The therapeutic effects of MSCs for AAA are mainly attributed to their immunomodulatory capacity. Nonetheless, this immunomodulatory capacity is largely affected by a patient's comorbid conditions. It has been reported that the immunomodulatory capacity of ASCs isolated from patients with atherosclerosis and type 2 diabetes mellitus is dramatically reduced [42]. MSCs isolated from atherosclerosis patients secrete high levels of IL-6, IL-8, and MCP-1, leading to a reduced immunomodulatory capacity [43]. Consistently, in the current study, we also observed higher levels of IL-6 and TNF-*α* and a lower level of IL-10 in the AAA-ASC secretome than in the H-ASC secretome, indicating a reduced immunopotency of AAA-ASCs. According to several studies, autophagy is closely associated with the secretion of inflammatory factors in various types of cells [44–46]. We also found that rapamycin treatment greatly downregulated the levels of IL-6 and TNF-*α* and upregulated the level of IL-10 in the AAA-ASC secretome, suggesting that autophagy mediates the secretion of inflammatory factors in AAA-ASCs, therefore affecting their immunomodulatory capacity.

There are several limitations in the current study that we need to acknowledge. First, although we found increased mitochondrial fusion in AAA-ASCs, whether mitochondrial fusion leads to AAA-ASC senescence requires further investigation. Second, we only analyzed three immunomodulatory cytokines in the secretome of AAA-ASCs. The alteration of other cytokines or factors in the AAA-ASC secretome remains unclear. Third, although we observed that AAA-ASCs secrete a higher level of several immunomodulatory cytokines, the immunomodulatory capacity of AAA-ASCs was not determined.

## 5. Conclusion

Our study shows that ASCs from AAA patients exhibit phenomena of senescence. In addition, we revealed impaired mitochondrial function and autophagy in AAA-ASCs. These results suggest that the therapeutic efficacy of AAA-ASCs may be impaired compared with H-ASCs. Targeting mitochondria or autophagy may enhance the therapeutic efficacy of ASCs derived from AAA patients in autologous cell-based therapy.

## Figures and Tables

**Table 1:** Demographic characteristics of the study subjects.

Total subjects	Control	AAA	*P* value
	11	13	—
Age (y), mean ± SEM	65.75 ± 5.509	64.9 ± 9.793	0.9377
Male (*n*, %)	8 (72.7%)	11 (84.6%)	—
Height (cm), mean ± SEM	168.1 ± 7.563	169.07 ± 7.444	0.7478
Weight (kg), mean ± SEM	64.1 ± 10.021	65.31 ± 9.358	0.7760
BMI (kg/m^2^), mean ± SEM	22.57 ± 2.148	22.71 ± 1.967	0.8704
BSA (m^2^), mean ± SEM	1.692 ± 0.171	1.797 ± 0.167	0.1591
Smoking (*n*, %)	2 (18.2%)	6 (46.1%)	—
Hypertension (*n*, %)	2 (18.2%)	7 (53.8%)	—

BMI: body mass index; BSA: body surface area; AAA: abdominal aortic aneurysm; BSA = 0.0061∗height (cm) + 0.0128∗weight (kg)–0.1529.

## Data Availability

The data used to support the findings of this study are included within the article.
